# Supplementary material for: Polyketide synthase gene clusters in the ericoid mycorrhizal fungus Oidiodendron maius: genome-wide prediction, expression profiling, and functional characterization of knockout mutants
Source: Front Plant Sci. 2026 Sep 16;17:1924430. doi: 10.3389/fpls.2026.1924430 (PMC13623903; doi:10.3389/fpls.2026.1924430)
Supplement: Supplementary file 1 [file SupplementaryFile1.zip › Supplementary Materials/Supplementary Folder 1.zip!/Oidma1_AssemblyScaffolds_Repeatmasked/knownclusterblast/cluster49/jgi.p_Oidma1_136671_mibig_hits.html]

| MIBiG Protein | Description | MIBiG Cluster | MiBiG Product | % ID | % Coverage | BLAST Score | E-value |
| --- | --- | --- | --- | --- | --- | --- | --- |
| AGN71605.1 | putative\_citrinin\_biosynthesis\_transcriptional\_activator\_CtnR | BGC0000027 | Azaphilone\_biosynthetic\_gene\_cluster | 40 | 88.7900355872 | 341 | 2e-93 |
| ALI92652.1 | MRL3\_Zn(II)2Cys6\_Transcriptional\_regulator | BGC0001338 | Citrinin\_biosynthetic\_gene\_cluster | 40 | 85.231316726 | 336 | 8.2e-92 |
| EHA28238.1 | hypothetical\_protein | BGC0001143 | Azanigerone\_biosynthetic\_gene\_cluster | 41 | 85.9430604982 | 318 | 1.8e-86 |
| CAP95402.1 |  | BGC0001404 | Sorbicillin\_biosynthetic\_gene\_cluster | 34 | 105.160142349 | 264 | 2.3e-70 |
| EED18008.1 | conserved\_hypothetical\_protein | BGC0000154 | Stipitatic\_acid\_biosynthetic\_gene\_cluster | 32 | 102.669039146 | 234 | 3.4e-61 |
| EAQ86389.1 | hypothetical\_protein | BGC0001405 | Chaetoviridin\_/\_chaetomugilin\_biosynthetic\_gene\_cluster | 35 | 73.8434163701 | 243 | 5.6e-64 |
| EAA65597.1 | hypothetical\_protein | BGC0000022 | Asperfuranone\_biosynthetic\_gene\_cluster | 35 | 80.9608540925 | 226 | 9.2e-59 |
